# Correction: Water distribution at the electrified interface of deep eutectic solvents

**DOI:** 10.1039/c9na90039j

**Published:** 2019-07-29

**Authors:** Mesfin Haile Mamme, Samuel L. C. Moors, El Amine Mernissi Cherigui, Herman Terryn, Johan Deconinck, Jon Ustarroz, Frank De Proft

**Affiliations:** Vrije Universiteit Brussel (VUB), Research Group Electrochemical and Surface Engineering (SURF) Pleinlaan 2 1050 Brussels Belgium mmamme@vub.be; Vrije Universiteit Brussel (VUB), Eenheid Algemene Chemie (ALGC) Pleinlaan 2 1050 Brussels Belgium; Vrije Universiteit Brussel (VUB), Department of Electrical Engineering and Energy Technology (ETEC) Pleinlaan 2 1050 Brussels Belgium; Université libre de Bruxelles, Chemistry of Surfaces, Interfaces and Nanomaterials (ChemSIN) Boulevard du Triomphe 2 1050 Brussels Belgium

## Abstract

Correction for ‘Water distribution at the electrified interface of deep eutectic solvents’ by Mesfin Haile Mamme *et al.*, *Nanoscale Adv.*, 2019, DOI: 10.1039/c9na00331b.

The authors regret the omission of one of the affiliations of the author Jon Ustarroz from the original manuscript. The correct affiliations for this paper are as shown.

The Royal Society of Chemistry apologises for these errors and any consequent inconvenience to authors and readers.

## Supplementary Material

